# Abnormal HDL lipid and protein composition following pediatric cancer treatment: an associative study

**DOI:** 10.1186/s12944-023-01822-2

**Published:** 2023-06-10

**Authors:** Véronique Bélanger, Sophia Morel, Mélanie Napartuk, Isabelle Bouchard, Caroline Meloche, Daniel Curnier, Serge Sultan, Caroline Laverdière, Daniel Sinnett, Valérie Marcil

**Affiliations:** 1 Research Centre, CHU Sainte-Justine, 3175 Chem. de la Côte-Sainte-Catherine, Montreal, QC H3T 1C5 Canada; 2 Department of Nutrition, Université de Montréal, Montreal, QC Canada; 3 School of Kinesiology and Physical Activity Sciences, Université de Montréal, Montreal, QC Canada; 4 Department of Psychology, Université de Montréal, Montreal, QC Canada; 5 Department of Pediatrics, Université de Montréal, Montreal, QC Canada

**Keywords:** Dyslipidemias, Lipoproteins, Apolipoproteins, High density lipoprotein, Cholesterol, Children, Adolescents, Chemotherapeutic agents, Clinical studies, Obesity

## Abstract

**Background:**

Long-term childhood cancer survivors (CCS) are at high risk of having dyslipidemia including low high density lipoprotein cholesterol (HDL-C). However, little is known about the prevalence of low HDL-C and the impact of therapy exposure on HDL composition early after treatment is terminated.

**Methods:**

This associative study included 50 children and adolescents who had completed their cancer treatments (< 4 years). Clinical characteristics (demographic, diagnosis, treatment, anthropometric parameters), fasting plasma lipids, apoliporoteins (Apo) A-I and composition of HDL fractions (HDL2 and HDL3) were assessed. Data were stratified according to the presence of dyslipidemia and median doses of therapeutic agents and compared using Fisher exact or Mann–Whitney tests. Univariate binary logistic regression analyses were carried out to evaluate the associations between the clinical and biochemical characteristics and having low HDL-C. Composition of HDL2 and HDL3 particles was assessed in a sub-group of 15 patients and compared to 15 age- and sex-matched healthy controls using Wilcoxon paired test.

**Results:**

Of the 50 pediatric cancer patients included in this study (mean age: 11.30 ± 0.72 y; mean time since end of treatment: 1.47 ± 0.12 y; male: 38%), 8 had low HDL-C (16%), all of which were adolescent at diagnosis. Higher doses of doxorubicin were associated with lower HDL-C and Apo A-I levels. In hypertriglyceridemic patients and compared to normolipidemics, triglycerides (TG) content was greater in HDL2 and HDL3 fractions whereas esterified cholesterol (EC) content was lower in HDL2. Enrich TG content of HDL3 and lower EC of HDL2 was found in patients exposed to ≥ 90 mg/m^2^ doxorubicin. Factors positively associated with the risk of having low HDL-C were age, being overweight or obese and exposure to doxorubicin ≥ 90 mg/m^2^. Compared to healthy controls, a sub-group of 15 patients showed higher TG and free cholesterol (FC) content of HDL2 and HDL3 and lower EC content in HDL3.

**Conclusions:**

Overall, we found abnormalities in HDL-C and Apo A-I levels and in HDL composition early after pediatric cancer treatment that are influenced by age, overweight or obesity status and exposure to doxorubicin.

**Supplementary Information:**

The online version contains supplementary material available at 10.1186/s12944-023-01822-2.

## Background

Due to the advances in pediatric cancer treatment, the 5-year survival rate in Canada is now reaching 84% for children 0 to 14 years old [1] and 86% in the 15 to 29 age group [2]. However, survivors of childhood and adolescent cancer may face severe long-term sequelae years after the end of treatments [3, 4]. Compared to their siblings or the general population, childhood cancer survivors (CCS) have an increased risk of cardiometabolic complications such as arterial hypertension, dyslipidemia, diabetes, and obesity [5–10]. These complications contribute to the higher risk of cardiovascular disease of CCS [11–13]. While cardiometabolic health after pediatric cancer has been described primarily in the long-term, i.e., over 5 years after diagnosis, a limited number of studies have evaluated it early after the end of treatments (< 5 years). The few studies that have investigated this issue support the presence of these health problems during this period [14–17]. Nonetheless, the mechanisms and time course related to the development of cardiometabolic complications between the end of treatment and survivorship remain nebulous.

Lipid abnormalities have been examined in CCS to appraise their cardiovascular risk. Mainly, studies reported high levels of triglycerides (TG) and low-density lipoprotein cholesterol (LDL-C), and low levels of high-density lipoprotein cholesterol (HDL-C) [5, 13]. However, it is well-known that atherogenic dyslipidemia is not solely reflected by one’s lipid profile: measuring LDL-C and HDL-C levels is important to assess cardiometabolic risk, but it appears that particle composition in lipids and proteins better predicts atherosclerosis [18, 19]. This has been highly documented for HDL particles, for which composition reflects functionality and atheroprotective properties [20–23]. A previous study by our group described, in a cohort of 80 young survivors of childhood acute lymphoblastic leukemia (ALL) (mean age 21.1 years, mean survival time 12.4 years), an altered lipid and apolipoprotein (Apo) composition of HDL2 particles when compared to a control group, suggesting defects in functionality [24, 25]. HDL particles are known for their ability to promote reverse cholesterol transport, but also for their antioxidant [26, 27], anti-apoptotic [28], anti-inflammatory [29], anti-hypertensive [30] and anti-thrombotic [31] roles.

Chemotherapeutic agents and radiation therapy have been associated with the development of cardiometabolic complications in CCS [5, 8, 32–38]. Among many, doxorubicin (an anthracycline) and methotrexate (an antimetabolite) are chemotherapeutic agents widely used to treat childhood cancer given their capacity to effectively damage cancer cells by inducing DNA hypermethylation [39]. However, they may also damage normal cells and thus accelerate the cellular aging process [40], a phenomenon that has been observed in young adult survivors of childhood ALL [41]. Of note, administration of doxorubicin and methotrexate has been associated with lipid disorders and cardiometabolic complications during and after treatment of breast cancer and childhood ALL [41–43]. Similarly, in children treated for ALL, administration of a five-day course of dexamethasone (a corticosteroid) during the maintenance phase resulted into an acute increase in HDL-C, total cholesterol (TC), LDL-C and TG levels [42].

There is a gap of knowledge on the fate of HDL levels and composition shortly after exposure to pediatric cancer treatment. Given the important role of HDL in cardiometabolic health and the development of cardiovascular disease, we need to increase knowledge regarding lipid metabolism disturbances of children and adolescents after cancer. This study aims at assessing the lipid and Apo profile of HDL fractions early after the end of pediatric cancer treatment (< 4 years) in comparison with healthy controls. The secondary objective is to determine the relationship between clinical (age at diagnosis, diagnosis, overweight or obesity status) and therapeutic parameters (exposure to doxorubicin, methotrexate and corticosteroids) and the lipid and Apo profile of HDL fractions in post-treatment pediatric cancer patients.

## Methods

### Study participants

Children and adolescents who had completed their cancer treatments were recruited from June 2017 to October 2019 as part of the VIE (Valorisation, Implication, Education) Study in the Division of Hematology-Oncology, CHU Sainte-Justine (CHUSJ) (Montreal, Canada). The study design was described elsewhere [16, 44]. The VIE Study is a multidisciplinary healthy lifestyle intervention (nutrition, physical activity, psychological support) in pediatric oncology. The present study includes only ‘control’ participants from the VIE study. In this regard, these participants had received a diagnosis of pediatric cancer, had completed their cancer treatment, were followed as outpatients at the Hematology-Oncology Division of the CHUSJ, but were not exposed to the VIE multidisciplinary lifestyle intervention. From these, a total of 50 participants were characterized for the current study and are referred to as post-treatment pediatric cancer patients. Assessments and measures were performed during a single visit that coincided with a medical follow-up appointment at the outpatient oncology clinic. Inclusion criteria were: 1) have received a diagnosis of pediatric cancer (≤ 21 years old) since less than 5 years; 2) have been treated with chemotherapy and/or radiotherapy and; 3) have completed cancer therapy. Patients with relapse or a second neoplasm were excluded from the study.

For comparison purposes, 15 unrelated healthy controls were recruited. Inclusion criteria for healthy controls were having no history of cancer and no known metabolic diseases including prediabetes or diabetes, dyslipidemia and arterial hypertension. Measures were collected during a single visit. Each healthy control was matched with one cancer participant for sex and age, with a margin of plus or minus 1 year, for a total of 15 pairs.

The study was approved by the Ethics Review Board of the CHUSJ (#2017–1413) and conducted in accordance with the Declaration of Helsinki. Written informed consent was obtained from each participant or parent/legal guardian.

### Demographic, diagnosis, and treatment characteristics of cancer participants

Information on participants’ demographic (sex and age at evaluation), diagnosis (age at diagnosis, cancer type and time since diagnosis), treatment [duration, time since end of treatment, exposure to radiotherapy (yes/none), exposure to doxorubicin (cumulative dose), methotrexate (cumulative dose) and corticosteroids (cumulative doses)] was collected from medical records. As previously stated [16], patients were grouped according to their age at diagnosis based on the WHO definition of adolescence in which ‘children’ refers to patients diagnosed < 10 years old, whereas ‘adolescents’ includes those diagnosed at ≥ 10 years [45].

### Anthropometric assessments of cancer participants and healthy controls

For post-treatment pediatric cancer patients and healthy controls, body weight and height were measured at the nearest 0.1 kg and 0.1 cm while standing without shoes and in light clothing using a calibrated electronic scale and a stadiometer, respectively. Body mass index (BMI) was calculated as weight (kg) / height (m)^2^. BMI-for-age was obtained using the Microsoft® Office Excel tool developed by the British Columbia Children’s Hospital and the Canadian Pediatric Endocrine Group based on the 2014 version of Growth Charts for Canada [46]. Being overweight or obese was defined as participants having BMI-for-age z-score > 2.00 for children aged between 2 and 5 years old [47] and > 1.00 z-score for participants > 5 years old [47–50].

### Biochemical analyses

For cancer patients and controls, 12 h overnight fasting blood was collected in tubes containing 1 g/L EDTA and was kept on ice until centrifugation. Plasma was separated by centrifugation [893 g, 20 min, 7 °C with an Avanti HP-30I centrifuge and JS 7.5 rotor, Beckman Coulter (Brea, CA, USA)] within 45 min of collection and stored at − 80 °C until analysis. TC, TG and HDL-C concentrations were measured, and associated z-scores for age and sex were calculated based on the available data of the Canadian Health Measured Survey (CHMS) Cycles 1 to 3 [51] as described previously [16]. LDL-C was calculated using the Friedewald formula [52]. Apolipoprotein A-I (Apo A-I) was quantified in plasma with an automated analyzer (Cobas Integra 400; Roche Diagnostics).

### HDL isolation

Lipoprotein separation was carried out as previously described [53–55]. Briefly, plasma was separated by low-speed centrifugation (893 g, 20 min) at 7 °C and the lipoprotein fractions were isolated by sequential ultracentrifugation with a 70.1Ti rotor in a Beckman Coulter Model LE-80 ultracentrifuge. After removal of LDL (1.063 g/ml) by centrifugation at 146 682 g for 18 h at 4 °C, isolation of HDL2 (1.125 g/ml) and HDL3 (1.21 g/ml) was carried out at 146 682 g for 48 h at 4 °C. Aliquots of HDL2 and HDL3 were stored at -80 °C until further analysis.

### Characterization of lipoprotein composition

The total protein content of each lipoprotein was determined by the Bio-Rad Bradford method with bovine serum albumin as a standard whereas phospholipids (PL) were measured using the Bartlett method [56]. Commercial kits were used to quantify TG (Randox TRIGS, UK), TC and free cholesterol (FC) (Wako Diagnostics, USA) by enzymatic colorimetric methods. Absorbance readings were gathered with the SpectraMax i3x plate reader. Esterified cholesterol (EC) was calculated as the difference between TC and FC multiplied by 1.6. Lipoprotein composition was determined as percentage of TG, EC, FC, PL and total protein in the 50 cancer patients and the 15 healthy controls.

The lipoprotein distribution of Apos was examined by electrophoresis on 4—20% gradient SDS-PAGE (4 – 20% Mini-PROTEAN TGX Stain-Free Precast Gels, 10 wells, 50 $\mu$ L) [53–55] in a sub-group of 15 cancer patients and 15 healthy controls, matched for age and sex. Apo densitometric distribution was assessed using the ChemiDoc MP & Imaging System (Bio-Rad, USA) for band visualization and analysis. Distribution of Apos was calculated as percentage of total Apos.

### Assessment of dyslipidemia

Dyslipidemia was determined by presenting at least one of three factors: high LDL-C, high TG, and/or low HDL-C. Lipid abnormalities were assessed based on current guidelines. TC values > 5.17 mmol/L in children and adolescents (0 – 19 years old) and > 5.79 mmol/L in 20 – 24 years old were considered high [57]. High TG were levels > 1.12 mmol/L in children 0 to 9 years old, > 1.46 mmol/L in children 10 years and over and > 1.69 mmol/L in 20 – 24 years old [57]. For LDL-C, values of ≥ 3.36 mmol/L in children and adolescents (0 – 19 years old) and ≥ 4.14 mmol/L (> 19 years old) were considered high. For all participants, levels of HDL-C < 1.03 mmol/L were considered low [57].

### Statistical analyses

Clinical and biochemical characteristics of cancer patients are portrayed using descriptive statistics. Continuous variables are expressed as means ± standard error of mean (SEM) unless otherwise specified, while categorical variables are presented as prevalence (%). Exposure to therapeutic agents was classified by doses received, categorized as below or equal and above the median. Lipid composition and total protein content of HDL2 and HDL3 were computed as percentage of TG, EC, FC, PL and total protein. Data were stratified according to the presence of dyslipidemia (yes / no) and median doses of therapeutic agents (low / high). Groups were compared using Mann–Whitney tests. Fisher exact tests were performed to assess the associations between therapeutic agents and having low HDL-C or dyslipidemia in pediatric cancer patients. Mann–Whitney tests were used to compare plasma HDL-C and Apo A-I concentrations between those who received low and high doses of therapeutic agents. Univariate binary logistic regression analyses were carried out to evaluate the associations between the clinical and biochemical characteristics and having low HDL-C.

Clinical and biochemical characteristics of the sub-group of 15 pediatric cancer patients and the 15 age- and sex-matched healthy controls are described using descriptive statistics. Wilcoxon paired-tests (age at evaluation, BMI-for-age, z-score, total cholesterol, TG, LDL-C, HDL-C) and Fisher exact tests (overweight or obese, dyslipidemia, hypercholesterolemia, hypertriglyceridemia, high LDL-C, low HDL-C) were performed to compare clinical and biochemical characteristic between groups. Lipid composition and total protein content of HDL2 and HDL3 were computed as percentage of TG, EC, FC, PL and total protein. Data were stratified according to the presence (yes/no) of dyslipidemia, hypertriglyceridemia and low HDL-C and compared with the Wilcoxon paired test. *P* < 0.05 was considered statistically significant. Statistical analyses were performed with IBM SPSS Statistics software (Version 27.0).

## Results

### Characteristics of post-treatment pediatric cancer patients

Sufficient plasma for HDL composition analysis was available for a total of 50 cancer patients (33 children and 17 adolescents). Their demographic and clinical characteristics are shown in Table 1. Mean age at evaluation was 11.30 years (range: 4.73 – 20.98 years), 62% (*n* = 31) were female and 54% (*n* = 27) had a diagnosis of leukemia. The average time since diagnosis was 3.07 years and the time since completion of treatment was 1.47 year, with an average treatment duration of 1.59 year. The median doses of doxorubicin, corticosteroids and methotrexate were 90 mg/m^2^, 8,080 mg/m^2^ and 7,230 mg/m^2^ respectively. Twenty-four percent of participants received radiotherapy at a mean dose of 33.5 Gy (range: 10.8 – 55.8 Gy). Of these, 7 patients had head and neck-oriented radiotherapy (mean 36.3 Gy; range: 12 – 54 Gy) and 5 received chest (mediastinal, *n* = 2), abdominal (*n* = 1) and pelvic (*n* = 2) radiotherapy with a mean dose of 29.6 Gy (range: 10.8 – 55.8 Gy).

**Table 1:** Clinical characteristics of post-treatment pediatric cancer patients

**Characteristics**	**Post-treatment pediatric cancer patients**
	*n* = 50
**Sex, n (%)**	
Male	19 (38)
Female	31 (62)
**Age group at diagnosis, n (%)**^**a**^	
Children	33 (66)
Adolescents	17 (34)
**Age at evaluation, y**	
Mean ± SEM	11.30 ± 0.72
Median (Min–Max)	8.95 (4.73 – 20.98)
**Time since diagnosis, y**	
Mean ± SEM	3.07 ± 0.09
Median (Min–Max)	3.03 (2.08 – 4.29)
**Time since end of treatment, y**	
Mean ± SEM	1.47 ± 0.12
Median (Min–Max)	1.51 (0.18 – 3.53)
**Treatment duration, y**	
Mean ± SEM	1.59 ± 0.11
Median (Min–Max)	2.09 (0.20 – 3.18)
**Diagnosis, n (%)**	
ALL^b^	27 (54)
Lymphoma^c^	11 (22)
Other^d^	12 (24)
**Doxorubicin dose, mg/m**^**2**^	
Mean ± SEM	147.5 ± 16.3
Median (Min–Max)	90.0 (0 – 450.0)
Doxorubicin < 90 mg/m^2^, n (%)	25 (50)
Doxorubicin ≥ 90 mg/m^2^, n (%)	25 (50)
**Corticosteroid dose, mg/m**^**2**^	
Mean ± SEM	5,939 ± 643
Median (Min–Max)	8,080 (0 – 12,680)
Corticosteroids < 8,080 mg/m^2^, n (%)	20 (40)
Corticosteroids ≥ 8,080 mg/m^2^, n (%)	30 (60)
**Methotrexate dose, mg/m**^**2**^	
Mean ± SEM	4,571 ± 545
Median (Min–Max)	7,230 (0 – 12,000)
Methotrexate < 7,230 mg/m^2^, n (%)	21 (42)
Methotrexate ≥ 7,230 mg/m^2^, n (%)	29 (58)
**Radiotherapy, n (%)**	
Yes	12 (24)
No	38 (76)
**BMI-for-age, z-score**	
Mean ± SEM	0.48 ± 0.17
Median (min–max)	0.28 (-1.37 – 3.49)
**Overweight or obese, n (%)**	
Yes	15 (30)
No	35 (70)

*SEM* standard error of the mean, *y* years

^a^Age groups at diagnosis were defined as adolescents: ≥ 10 years and children: < 10 years

^b^Includes acute lymphoblastic leukemia (*n* = 26) and B mature leukemia with mixed-lineage leukemia (*n* = 1)

^c^Includes non-Hodgkin lymphoma (*n* = 5) and Hodgkin’s lymphoma (*n* = 6)

^d^Other diagnoses include: rhabdomyosarcoma (*n* = 1), Wilm’s tumor (*n* = 2), neuroblastoma (*n* = 2), synovial sarcoma (*n* = 1), medulloblastoma (*n* = 1) and histiocytosis (*n* = 1), Ewing’s sarcoma (*n* = 1), rhabdomyosarcoma (*n* = 1), oligodendroglioma (*n* = 1), osteosarcoma (*n* = 1)

Comparisons based on age at diagnosis (< 10 vs. ≥ 10 years old) revealed differences in clinical characteristics in terms of diagnoses, doses of doxorubicin and methotrexate (Supplementary Table 1). The predominant diagnosis in children was leukemia (69.7%), while half of adolescents had lymphoma. On average, adolescents received higher doses of doxorubicin (255.0 ± 23.9 mg/m^2^) than children (93.7 ± 14.3 mg/m^2^), whereas children received higher doses of corticosteroids (6,589 ± 708 mg/m^2^) and methotrexate (5,616 ± 597 mg/m^2^) than adolescents (4,676 ± 1,274; 2,543 ± 951 mg/m^2^). This is reflected by more adolescents than children having received ≥ 90 mg/m^2^ of doxorubicin (16/17 vs. 9/33; *p* < 0.001), and more children having received ≥ 8,080 mg/m^2^ of corticosteroids (24/33 vs. 6/17; *p* = 0.015) and ≥ 7,230 mg/m^2^ of methotrexate (24/33 vs. 5/17; *p* = 0.006). No difference in the number of patients treated with radiotherapy or in the proportion of overweight/obesity between groups was observed.

### Dyslipidemia in post-treatment pediatric cancer patients

Thirty percent (*n* = 15) of the 50 participants had dyslipidemia, 10% (*n* = 5) had hypercholesterolemia, 10% (*n* = 5) high LDL-C, 12% (*n* = 6) hypertriglyceridemia and 16% (*n* = 8) low HDL-C (Table 2). Of note, three participants had a combined form of dyslipidemia, one had high TG and low HDL-C, one had high LDL-C and low HDL-C and one had high TG, high LDL-C and low HDL-C. All 8 patients with low HDL-C were adolescents; no child had low HDL-C (Supplementary Table 2). Of the 8 adolescents with low HDL-C, 7 had received high doses (≥ 90 mg/m^2^) of doxorubicin. Mean HDL-C levels and z-scores were higher in children than in adolescents (1.45 ± 0.04 vs. 1.15 ± 0.06 mmol/L; *p* < 0.001 and 0.16 ± 0.15 vs. -0.64 ± 0.20; *p* = 0.002). Mean plasma Apo A-I concentrations followed the same trend (1.44 ± 0.03 vs. 1.28 ± 0.06; *p* = 0.002).

**Table 2:** Plasma lipid profile of post-treatment pediatric cancer patients

	**Post-treatment pediatric cancer patients**
**Dyslipidemia, n (%)**	
Yes	15 (30.0)
No	35 (70.0)
**Cholesterol total**	
**mmol/L**	*n* = 50
Mean ± SEM	4.10 ± 0.11
Median (Min–Max)	4.01 (2.27 – 6.09)
**z-score**	*n* = 45
Mean ± SEM	-0.07 ± 0.16
Median (Min–Max)	-0.20 (-2.62 – 2.24)
**Hypercholesterolemia, n (%)**	
Yes	5 (10.0)
No	45 (90.0)
**TG**	
**mmol/L**	*n* = 50
Mean ± SEM	0.82 ± 0.06
Median (Min–Max)	0.71 (0.25 – 2.34)
**z-score**	*n* = 45
Mean ± SEM	-0.15 ± 0.16
Median (Min–Max)	-0.17 (-2.71 – 2.42)
**Hypertriglyceridemia, n (%)**	
Yes	6 (12.0)
No	44 (88.0)
**LDL-C, mmol/L**	*n* = 50
Mean ± SEM	2.38 ± 0.10
Median (Min–Max)	2.36 (0.60 – 4.23)
**High LDL-C, n (%)**	
Yes	5 (10.0)
No	45 (90.0)
**HDL-C**	
**mmol/L**	*n* = 50
Mean ± SEM	1.35 ± 0.04
Median (Min–Max)	1.34 (0.85 – 2.01)
**z-score**	*n* = 45
Mean ± SEM	-0.14 ± 0.13
Median (Min–Max)	-0.20 (-2.13 – 1.88)
**Low HDL-C, n (%)**	
Yes	8 (16.0)
No	42 (84.0)
**Apo A-I, mmol/L**	*n* = 50
Mean ± SEM	1.38 ± 0.03
Median (Min–Max)	1.36 (0.98 – 1.91)

Dyslipidemia was defined when participants presented at least one of three factors: high LDL-C, high TG, and/or low HDL-C. In children 0 to 9 years old, TG > 1.12 mmol/L, in adolescents 10 to 19 years old, TG > 1.46 mmol/L and in participants 20 – 24 years old, TG > 1.69 mmol/L were considered high. For LDL-C, values of ≥ 3.36 mmol/L in participants 0 – 19 years old and values ≥ 4.4 mmol/L in > 19 years old were considered high. Levels of HDL-C < 1.03 mmol/L in all participants were considered low. Apo A-I: apolipoprotein A-I; *SEM* standard error of the mean, *y* years

**Fig. 1:**
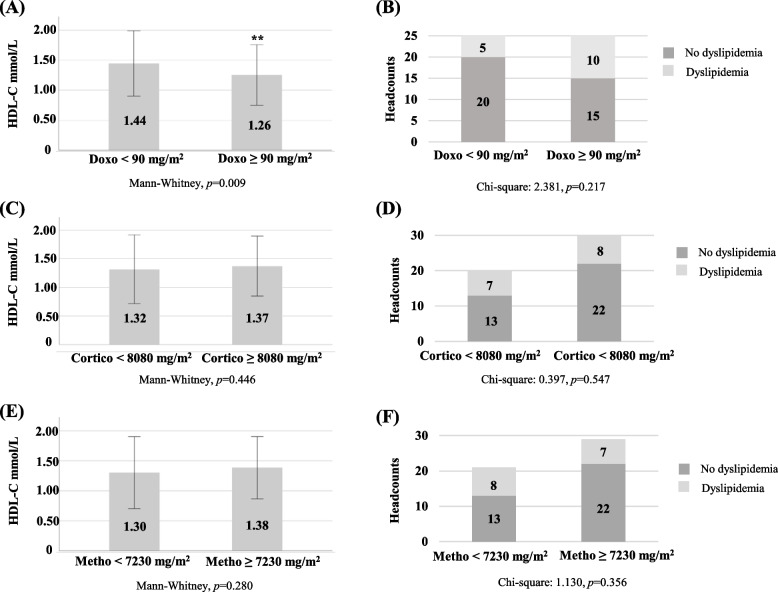
Comparison of selected lipid variables between patients who received low and high doses of therapeutic agents. Plasma levels of (**A**) HDL-C; **B** Apo A-I; and proportions of (**C**) dyslipidemia; and (**D**) low HDL-C were compared between patients according to their exposure to doxorubicin, methotrexate and corticosteroids. Cumulative doses were classified as low vs. high for doxorubicin (< 90 mg/m^2^ vs. ≥ 90 mg/m^2^). methotrexate (< 7,230 mg/m^2^ vs. ≥ 7,230 mg/m^2^) and corticosteroids (< 8,080 mg/m^2^ vs. ≥ 8,080 mg/m^2^). Data (**A** and **B**) are expressed as mean ± SEM and as (**C** and **D**) percentage of patients. **p-value < 0.01 for difference between low vs. high doses according to Mann–Whitney test. *p-value < 0.05 for differences between low vs. high doses according to Chi-Square test

Figure 1 illustrates the comparison in plasma levels of HDL-C and Apo A-I and in the proportion of dyslipidemia and of low HDL-C between patients who received low and high doses of therapeutic agents. Patients who had received ≥ 90 mg/m^2^ of doxorubicin had lower HDL-C concentrations and higher prevalence of low HDL-C than those who had < 90 mg/m^2^ (Figs. 1A and D). Although not statistically significant, there was a trend for lower Apo A-I concentrations and a higher percentage of dyslipidemia for patients who received ≥ 90 mg/m^2^ of doxorubicin compared to those who received smaller doses (Figs. 1B and C). No difference was observed when patients were grouped according to whether they received low or high doses of corticosteroids and methotrexate (Figs. 1A, B, C and D).

### Composition of lipid and protein moieties of HDL2 and HDL3 in post-treatment pediatric cancer patients

#### According to lipidemia status

The main difference in lipid and protein composition of HDL2 and HDL3 between patients with and without dyslipidemia was the relative TG content (Table 3). In patients with hypertriglyceridemia, both HDL fractions were enriched in TG. Comparing HDL2 and HDL3 of patients according to age groups showed that the relative TG content in HDL3 particles was higher in adolescents than in children (Supplementary Table 3). Differences between age groups were also observed in the percentage of EC content in HDL2 from dyslipidemic patients and in HDL3 from normolipidemics. The weight ratio estimates the size of lipoproteins by evaluating the mass ratio of core constituents (TG + EC) to surface constituents (FC + PR + PL), as lighter and larger particles are relatively enriched with TG and/or EC [53]. Whereas the weight ratio of HDL2 and HDL3 was not different between dyslipidemic adolescents and children, HDL3 weight ratio was higher in patients who were diagnosed ≥ 10 years old compared to younger (Supplementary Table 3).

**Table 3:** Composition of HDL2 and HDL3 moieties of post-treatment pediatric cancer patients with or without dyslipidemia

	**TG**	**FC**	**EC**	**PL**	**PR**	**Weight ratio** [(TG + EC):(FC + PL + PR)]
**HDL2** (1.125 g/mL)						
Total, *n* = 50	2.67 ± 0.24	5.01 ± 0.18	12.96 ± 0.54	30.26 ± 0.82	49.1 ± 0.70	0.19 ± 0.01
No dyslipidemia, *n* = 35	2.43 ± 0.24	4.88 ± 0.21	13.44 ± 0.64	29.22 ± 0.60	50.02 ± 0.63	0.19 ± 0.01
Dyslipidemia, *n* = 15	3.22 ± 0.59	5.33 ± 0.36	11.84 ± 0.85	32.69 ± 2.29	46.93 ± 1.71	0.18 ± 0.01
Low HDL-C, *n* = 8	2.92 ± 0.83	5.53 ± 0.48	11.55 ± 1.37	34.60 ± 4.26	45.41 ± 3.11	0.17 ± 0.02
Hypertriglyceridemia, *n* = 6	4.76 ± 1.03*	5.26 ± 0.66	9.75 ± 1.61*	32.57 ± 2.93	47.66 ± 1.94	0.17 ± 0.02
**HDL3** (1.21 g/mL)						
Total, *n* = 50	1.63 ± 0.11	1.80 ± 0.09	10.96 ± 0.31	24.07 ± 0.55	61.54 ± 0.74	0.14 ± 0.00
No dyslipidemia, *n* = 35	1.46 ± 0.11	1.75 ± 0.08	10.88 ± 0.39	23.81 ± 0.73	62.09 ± 0.99	0.14 ± 0.01
Dyslipidemia, *n* = 15	2.01 ± 0.23*	1.90 ± 0.21	11.15 ± 0.55	24.68 ± 0.69	60.25 ± 0.85	0.15 ± 0.01
Low HDL-C, *n* = 8	1.85 ± 0.21	1.87 ± 0.17	11.84 ± 0.39	24.43 ± 1.17	60.01 ± 1.38	0.16 ± 0.01
Hypertriglyceridemia, *n* = 6	2.81 ± 0.29***	1.87 ± 0.52	9.78 ± 1.08	24.56 ± 0.59	60.98 ± 1.00	0.14 ± 0.01

Data (mean ± SEM) are expressed as percentage of total HDL2 and HDL3 content of *n* = 50 post-treatment pediatric cancer patients. Patients were stratified in two groups according to their dyslipidemia status as described in Materials and Methods. Two additional subgroups were stratified among dyslipidemic patients: individuals with low HDL (*n* = 8) and hypertriglyceridemic individuals (*n* = 6). Groups with dyslipidemia, low HDL-C or hypertriglyceridemia were compared to the group without dyslipidemia using Mann–Whitney tests. *p < 0.05, **p < 0.01, ***p < 0.001 vs. patients without dyslipidemia. *FC* free cholesterol, *EC* esterified cholesterol, *PL* phospholipids, *PR* protein, *SEM* standard error of the mean

#### According to exposure to therapeutic agents

**Fig. 2:**
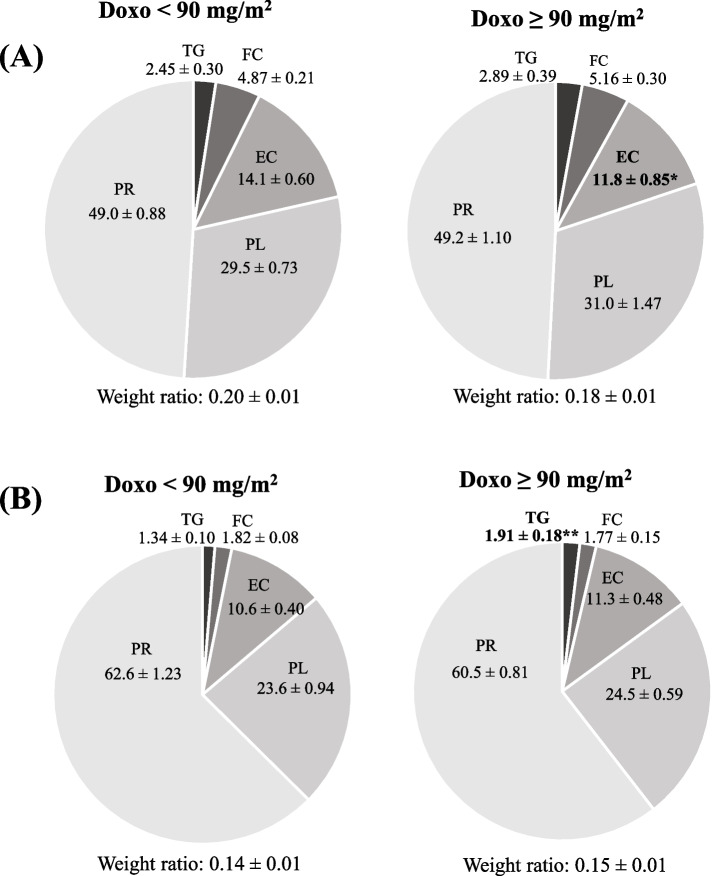
HDL2 and HDL3 composition of patients after pediatric cancer according to doses of doxorubicin received. Data of (**A**) HDL2 and (**B**) HDL3 were compared between patients treated with doxorubicin < 90 mg/m^2^ and ≥ 90 mg/m.^2^. Data are expressed as percentage of total lipoprotein content (mean ± SEM). SEM, standard error of the mean. *p < 0.05, **p < 0.01

The comparison of the lipid and protein content of HDL2 and HDL3 based on exposure to chemotherapeutic doses of doxorubicin is presented in Fig. 2. A higher proportion of TG was found in HDL3 of patients treated with doses ≥ 90 mg/m^2^, whereas the proportion of EC was lower in HDL2 of the same group. Besides, in HDL2, the proportion of FC was lower in patients treated with doses of methotrexate ≥ 7,230 mg/m^2^ (data not shown in Figure). No other difference was observed according to doses of doxorubicin, nor of corticosteroid and methotrexate (data not shown).

### Association between clinical and biochemical characteristics and having low HDL-C in post-treatment pediatric cancer patients

Univariate binary logistic regression analyses were performed to evaluate the associations between age at diagnosis, age at evaluation, doxorubicin ≥ 90 mg/m^2^, doxorubicin dose, overweight or obesity status and having low HDL-C (Table 4). All five independent variables were found positively associated with the risk of having low HDL-C.

**Table 4:** Association between age at evaluation, doses of doxorubicin, overweight or obesity status and having low HDL-C in post-treatment pediatric cancer patients

Low HDL-C	Odds Ratio	95% CI	*p*-value
Age at diagnosis (years)	1.44	1.12 – 1.86	0.004
Age at evaluation (years)	1.43	1.12 – 1.82	0.004
Doxorubicin ≥ 90 mg/m^2^	9.33	1.05 – 82.7	0.045
Doxorubicin dose, mg/m^2^	1.018	1.001 – 1.02	0.026
Overweight or obese	11.00	1.89 – 64.06	0.008

Binary logistic regression analyses (univariate model) were performed to define the association between age at evaluation (years), treatment with ≥ 90 mg/m^2^ doxorubicin, doxorubicin dose (mg/m^2^), being overweight or obese, and having low HDL-C. CI: confidence interval

### Comparisons between a sub-group of post-treatment pediatric cancer patients and healthy controls

The characteristics of the pairs (*n* = 15) are presented in Table 5. By comparing healthy controls to the sub-group of post-treatment pediatric cancer patients, there was no difference in mean age (12.9 vs. 12.6 years), mean BMI-for-age (0.13 vs. 0.50 z-score) and proportion of overweight/obesity (20.0% vs. 33.3%).

**Table 5:** Clinical characteristics of pairs of post-treatment cancer patients and age- and sex-matched controls

Characteristics	Healthy controls	Post-treatment pediatric cancer patients	*p*-value
	*n* = 15	*n* = 15	
**Sex, n (%)**			1.000
Male	8 (53.3)	8 (53.3)	
Female	7 (46.7)	7 (46.7)	
**Age group at diagnosis**			
Children < 10 yrs	N/A	8 (53.3)	
Adolescents ≥ 10 yrs		7 (46.7)	
**Age at evaluation, y**			0.838
Mean ± SEM	12.9 ± 1.42	12.6 ± 1.42	
Median (Min–Max)	11.9 (4.9 – 21.2)	10.9 (5.6 – 20.9)	
**Time since diagnosis, y**			
Mean ± SEM	N/A	2.9 ± 0.11	
Median (Min–Max)		2.85 (2.3 – 3.7)	
**Time since end of treatment, y**			
Mean ± SEM	N/A	1.65 ± 0.25	
(Min–Max)		2.1 (0.3 – 3.1)	
**Treatment duration, y**			
Mean ± SEM	N/A	1.25 ± 0.22	
Median (Min–Max)		1.0 (0.2 – 2.1)	
**Diagnosis, n (%)**			
ALL	N/A	7 (46.7)	
Other^a^		8 (53.3)	
**Doxorubicin dose, mg/m**^**2**^			
Mean ± SEM	N/A	158.7 ± 29.8	
Median (Min–Max)		135 (0 – 375)	
**Doxorubicin ≥ 90 mg/m**^**2**^			
Yes	N/A	9 (60)	
No		6 (40)	
**Radiotherapy, n (%)**			
Yes	N/A	3 (20.0)	
No		12 (80.0)	
**BMI-for-age, z-score**			0.489
Mean ± SEM	0.13 ± 0.27	0.50 ± 0.36	
Median (min–max)	0.15 (-1.95 – 1.85)	0.24 (-1.37 – 2.82)	
**Overweight or obese, n (%)**			0.682
Yes	3 (20.0)	5 (33.3)	
No	12 (80.0)	10 (66.7)	

A subgroup of cancer patients (*n* = 15) was matched for age and sex with healthy controls (*n* = 15) with no history of cancer or metabolic diseases. Pearson Chi-square (sex), Fisher exact (overweight or obese) and Mann–Whitney (age at evaluation, BMI-for-age, z-score) tests were performed to compare clinical characteristics between groups

*ALL* acute lymphoblastic leukemia, *N/A* non-applicable, *SEM* standard error of the mean, *y* years

^a^Other diagnoses include: non-Hodgkin lymphoma (*n* = 2), Hodgkin’s lymphoma (*n* = 3), rhabdomyosarcoma (*n* = 1), Wilm’s tumor (*n* = 1), Ewing’s sarcoma (*n* = 1)

Comparison of plasma lipid profiles revealed no statistically significant difference between groups (Table 6).

**Table 6:** Comparison of plasma lipid profile between post-treatment cancer patients and age- and sex-matched controls

Parameters	Healthy controls	Post-treatment pediatric cancer patients	*p*-value
**Dyslipidemia, n (%)**			0.107
Yes	2 (13.3)	6 (40.0)	
No	13 (86.7)	9 (60.0)	
**Total cholesterol**			
**mmol/L**	*n* = 15	*n* = 15	0.628
Mean ± SEM	3.90 ± 0.14	4.06 ± 0.20	
Median (Min–Max)	3.78 (2.93 – 5.04)	4.05 (2.27 – 5.47)	
**z-score**	*n* = 14	*n* = 13	0.542
Mean ± SEM	-0.34 ± 0.19	-0.12 ± 0.32	
Median (Min–Max)	-0.52 (-1.46 – 1.18)	-0.03 (-2.62 – 1.73)	
**Hypercholesterolemia, n (%)**			1.000
Yes	0	1 (6.7)	
No	15 (100)	14 (93.3)	
**TG**			
**mmol/L**	*n* = 15	*n* = 15	0.203
Mean ± SEM	0.72 ± 0.04	0.97 ± 0.14	
Median (Min–Max)	0.71 (0.44 – 1.02)	0.77 (0.46 – 2.34)	
**z-score**	*n* = 14	*n* = 13	0.146
Mean ± SEM	-0.33 ± 0.14	0.29 ± 0.30	
Median (Min–Max)	-0.32 (-1.59 – 0.64)	-0.01 (-1.34 – 2.42)	
**Hypertriglyceridemia, n (%)**			0.224
Yes	0	3 (20)	
No	15 (100)	12 (80)	
**LDL-C, mmol/L**	*n* = 15	*n* = 15	0.793
Mean ± SEM	2.20 ± 0.11	2.11 ± 0.18	
Median (Min–Max)	2.15 (1.54 – 3.16)	2.15 (0.60 – 3.57)	
**High LDL-C, n (%)**			1.000
Yes	0	1 (6.7)	
No	15 (100)	14 (93.3)	
**HDL-C**			
**mmol/L**	*n* = 15	*n* = 15	0.934
Mean ± SEM	1.38 ± 0.07	1.36 ± 0.08	
Median (Min–Max)	1.44 (0.95 – 1.71)	1.30 (0.97 – 2.01)	
**z-score**	*n* = 14	*n* = 13	0.721
Mean ± SEM	-0.02 ± 0.18	-0.22 ± 0.21	
Median (Min–Max)	0.12 (-1.01 – 0.89)	-0.23 (-1.34 – 1.10)	
**Low HDL-C, n (%)**			1.000
Yes	2 (13.3)	3 (20)	
No	13 (86.7)	12 (80)	

Dyslipidemia was defined when participants presented at least one of three factors: high LDL-C, high TG, and/or low HDL-C. In children 0 to 9 years old, TG > 1.12 mmol/L, in adolescents 10 to 19 years old, TG > 1.46 mmol/L and in participants 20 – 24 years old, TG > 1.69 mmol/L were considered high. For LDL-C, values of ≥ 3.36 mmol/L in participants 0 – 19 years old and values ≥ 4.4 mmol/L in > 19 years old were considered high. Levels of HDL-C < 1.03 mmol/L in all participants were considered low. Fisher exact (dyslipidemia, hypercholesterolemia, hypertriglyceridemia, high LDL-C and low HDL-C) and Wilcoxon paired-test (total cholesterol, TG, LDL-C, HDL-C) were performed to compare cancer patients and healthy controls. *SEM* standard error of the mean, *y* years

After analyzing HDL2 and HDL3 composition, we found higher proportions of TG and FC in both HDL fractions in patients compared to controls (Table 7). The proportion of EC was lower in patients than in controls, but the difference was found statistically significant only in HDL3. Moreover, HDL3 weight ratio was lower in patients.

**Table 7:** Comparison of HDL2 and HDL3 composition between post-treatment cancer patients and age- and sex-matched controls

	**TG**	**FC**	**EC**	**PL**	**PR**	**Weight ratio** [(TG + EC):(FC + PL + PR)]
**HDL2** (1.125 g/mL)						
Patients *n* = 15	3.50 ± 0.49**	5.30 ± 0.45*	13.04 ± 1.28	29.46 ± 0.96	48.70 ± 0.96	0.20 ± 0.02
No dyslipidemia, *n* = 9	2.68 ± 0.30	5.12 ± 0.68	14.42 ± 1.52	29.94 ± 1.49	47.84 ± 1.49	0.21 ± 0.02
Dyslipidemia, *n* = 6	4.73 ± 0.97	5.57 ± 0.52	10.96 ± 2.10	28.75 ± 0.98	49.99 ± 0.78	0.19 ± 0.03
Low HDL-C, *n* = 3	4.85 ± 1.26	4.80 ± 0.32	12.52 ± 2.91	28.82 ± 2.07	49.02 ± 1.31	0.21 ± 0.04
Hypertriglyceridemia, *n* = 3	6.60 ± 0.77	6.16 ± 1.00	7.67 ± 1.66	28.38 ± 0.93	51.19 ± 0.51	0.17 ± 0.03
Controls *n* = 15	2.14 ± 0.28	4.38 ± 0.18	15.96 ± 1.15	28.90 ± 0.79	48.87 ± 1.58	0.22 ± 0.02
No dyslipidemia, *n* = 13	1.85 ± 0.21	4.40 ± 0.20	16.40 ± 1.35	28.64 ± 0.86	48.98 ± 1.81	0.23 ± 0.02
Dyslipidemia, *n* = 2	4.04 ± 0.80	4.26 ± 0.67	13.06 ± 2.36	30.55 ± 1.96	48.10 ± 2.84	0.21 ± 0.02
Low HDL-C, *n* = 2	4.04 ± 0.80	4.26 ± 0.67	13.06 ± 2.36	30.55 ± 1.96	48.10 ± 2.84	0.21 ± 0.02
Hypertriglyceridemia, *n* = 0	N/A	N/A	N/A	N/A	N/A	N/A
**HDL3** (1.21 g/mL)						
Patients *n* = 15	1.84 ± 0.22*	1.83 ± 0.21**	10.91 ± 0.59***	23.93 ± 1.05	61.49 ± 1.27	0.15 ± 0.01***
No dyslipidemia, *n* = 9	1.48 ± 0.12	1.75 ± 0.13	11.19 ± 0.52	23.34 ± 1.58	62.24 ± 1.85	0.15 ± 0.01
Dyslipidemia, *n* = 6	2.37 ± 0.45	1.95 ± 0.50	10.50 ± 1.31	24.81 ± 1.19	60.38 ± 1.63	0.15 ± 0.01
Low HDL-C, *n* = 3	2.08 ± 0.41	1.41 ± 0.29	11.91 ± 0.63	25.38 ± 2.39	59.22 ± 3.02	0.16 ± 0.01
Hypertriglyceridemia, *n* = 3	3.21 ± 0.35	2.46 ± 0.94	8.67 ± 2.13	24.60 ± 1.06	61.05 ± 1.95	0.14 ± 0.03
Controls *n* = 15	1.25 ± 0.10	1.13 ± 0.08	14.70 ± 0.81	24.05 ± 0.77	57.24 ± 1.95	0.20 ± 0.01
No dyslipidemia, *n* = 13	1.19 ± 0.11	1.09 ± 0.09	14.73 ± 0.94	24.20 ± 0.88	56.90 ± 2.25	0.20 ± 0.01
Dyslipidemia, *n* = 2	1.64 ± 0.17	1.36 ± 0.19	14.49 ± 0.42	23.04 ± 0.96	59.49 ± 0.19	0.19 ± 0.01
Low HDL-C, *n* = 2	1.64 ± 0.17	1.36 ± 0.19	14.49 ± 0.42	23.04 ± 0.96	59.49 ± 0.19	0.19 ± 0.01
Hypertriglyceridemia, *n* = 0	N/A	N/A	N/A	N/A	N/A	N/A

Data (mean ± SEM) are expressed as percentage of total HDL2 and HDL3 content of 15 patients post pediatric cancer treatment and 15 age- and sex-matched controls. Patients and controls were stratified in two groups according to their dyslipidemia status as described in Materials and Methods. Two additional subgroups were stratified among dyslipidemic patients and controls: individuals with low HDL (patients, *n* = 3; controls, *n* = 2) and hypertriglyceridemic individuals (patients, *n* = 3; controls, *n* = 0). Wilcoxon paired-test were performed to compare cancer patients and healthy controls. *p < 0.05, **p < 0.01, ***p < 0.001 vs. healthy controls. HDL2: high-density lipoprotein 2 (density of 1.125 g/mL); HDL3: high-density lipoprotein 3 (density of 1.21 g/mL); TG: triglycerides; FC: free cholesterol; EC: esterified cholesterol; PL: phospholipids; PR: protein; SEM: standard error of mean

Table 8 summarizes the Apo composition of HDL2 and HDL3 in post-treatment pediatric cancer patients and healthy controls. Differences were found only in HDL2 particles, where there was increased Apo A-I and reduced Apo A-II proportions in patients versus controls. This resulted in a higher Apo A-I/A-II ratio in patients, but the difference did not reach statistical significance.

**Table 8:** Comparison of apolipoprotein moieties of HDL2 and HDL3 between post-treatment cancer patients and age- and sex-matched controls

Lipoprotein	Composition in apolipoproteins					Ratio
	E	A-I	C-III	C-II	A-II	A-I/A-II
	*Percentage of total content*					
**HDL2 (1.125 g/ml)**						
Patients *n* = 15	2.13 ± 0.16	76.67 ± 1.48*	12.56 ± 0.90	1.45 ± 0.14	7.18 ± 0.70**	12.20 ± 1.21
No dyslipidemia, *n* = 9	2.14 ± 0.25	76.66 ± 2.15	11.77 ± 1.14	1.49 ± 0.22	7.94 ± 0.98	11.20 ± 1.70
Dyslipidemia, *n* = 6	2.13 ± 0.19	76.69 ± 2.08	13.74 ± 1.43	1.39 ± 0.13	6.05 ± 0.80	13.70 ± 1.60
Low HDL-C, *n* = 3	1.82 ± 0.11	77.90 ± 4.33	12.39 ± 2.58	1.37 ± 0.25	6.52 ± 1.63	13.56 ± 3.24
Hypertriglyceridemia, *n* = 3	2.23 ± 0.34	77.73 ± 2.97	13.83 ± 2.55	1.25 ± 0.05	4.96 ± 0.23	15.76 ± 1.15
Controls *n* = 15	2.54 ± 0.33	72.49 ± 1.78	14.48 ± 0.76	1.87 ± 0.24	8.62 ± 0.88	10.47 ± 1.74
No dyslipidemia, *n* = 13	2.70 ± 0.36	71.16 ± 1.65	15.08 ± 0.72	1.99 ± 0.25	9.07 ± 0.90	9.00 ± 1.04
Dyslipidemia, *n* = 2	1.46 ± 0.49	81.14 ± 5.88	10.56 ± 1.82	1.11 ± 0.62	5.73 ± 2.96	20.02 ± 11.36
Low HDL-C, *n* = 2	1.46 ± 0.49	81.14 ± 5.88	10.56 ± 1.82	1.11 ± 0.62	5.73 ± 2.96	20.02 ± 11.36
Hypertriglyceridemia, *n* = 0	N/A	N/A	N/A	N/A	N/A	N/A
**HDL3 (1.210 g/ml)**						
Patients *n* = 15	-	83.39 ± 0.79	7.32 ± 0.37	0.82 ± 0.07	8.46 ± 0.55	10.37 ± 0.59
No dyslipidemia, *n* = 9	-	82.55 ± 1.11	7.19 ± 0.44	0.91 ± 0.08	9.35 ± 0.78	9.29 ± 0.71
Dyslipidemia, *n* = 6	-	84.66 ± 0.97	7.52 ± 0.71	0.69 ± 0.09	7.13 ± 0.28	11.99 ± 0.63
Low HDL-C, *n* = 3	-	84.09 ± 1.56	8.15 ± 1.14	0.74 ± 0.08	7.02 ± 0.37	12.07 ± 0.89
Hypertriglyceridemia, *n* = 3	-	84.79 ± 1.73	7.49 ± 1.37	0.61 ± 0.16	7.11 ± 0.45	12.06 ± 1.01
Controls *n* = 15	-	83.04 ± 0.86	8.10 ± 0.33	0.82 ± 0.09	8.04 ± 0.56	10.95 ± 0.67
No dyslipidemia, *n* = 13	-	82.71 ± 0.92	8.26 ± 0.34	0.85 ± 0.10	8.17 ± 0.63	10.76 ± 0.72
Dyslipidemia, *n* = 2	-	85.15 ± 2.32	7.08 ± 1.21	0.60 ± 0.01	7.17 ± 1.12	12.22 ± 2.23
Low HDL-C, *n* = 2	-	85.15 ± 2.32	7.08 ± 1.21	0.60 ± 0.01	7.17 ± 1.12	12.22 ± 2.23
Hypertriglyceridemia, *n* = 0	-	N/A	N/A	N/A	N/A	N/A

Data (mean ± SEM) are expressed as percentage of total HDL2 and HDL3 content of 15 patients after pediatric cancer treatment and 15 age- and sex-matched controls. Patients and controls were stratified in two groups according to their dyslipidemia status as described in Materials and Methods. Two additional subgroups were stratified in two groups among dyslipidemic patients and controls: hypertriglyceridemic individuals (patients, *n* = 3; controls, *n* = 0) and individuals with low HDL (patients, *n* = 3; controls, *n* = 2). Apo distribution was analysed using SDS-PAGE (4–20% gradient). Wilcoxon paired-test (cholesterol total, TG, LDL-C, HDL-C) were performed to compare cancer patients and healthy controls. HDL2: high-density lipoprotein 2 (density of 1.125 g/mL); HDL3: high-density lipoprotein 3 (density of 1.21 g/mL); SEM: standard error of mean. *p < 0.05, **p < 0.01, ***p < 0.001 vs. healthy controls

## Discussion

In our study, participating children and adolescents were evaluated shortly after the completion of their cancer treatment (mean time of 1.47 ± 0.12 years). We found that only adolescents had low HDL-C (8 out of 17 adolescents, 47.1%) and that most of them had received doses of doxorubicin ≥ 90 mg/m^2^ (7 out of 8, 87.5%). Higher doses of doxorubicin were associated with lower plasma HDL-C and Apo A-I levels, while exposure to corticosteroids and methotrexate had no impact. Compared to non dyslipidemic, TG content of hypertriglyceridemic patients was greater in HDL2 and HDL3 fractions whereas EC was lower in HDL2. Similarly, HDL2 of patients exposed to ≥ 90 mg/m^2^ of doxorubicin had lower EC and higher TG content in HDL3. Factors positively associated with the risk of having low HDL-C were age, being overweight or obese, and having received doses of doxorubicin ≥ 90 mg/m^2^. Finally, comparing a sub-group of 15 patients to healthy controls with similar potential cofounding characteristics (age, sex and overweight/obesity status) showed higher TG and FC content of HDL2 and HDL3 and lower EC content in HDL3. Overall, these results support the presence of abnormalities in HDL-C and Apo A-I profile and in HDL composition early after cancer treatment and the influence of age, overweight or obesity status and exposure to doxorubicin.

Low HDL-C levels have been reported at the time of ALL diagnosis and during maintenance therapy in children and adolescents [58–60]. However, outside our study, few data are available on the prevalence of low HDL-C between the end of treatment and long-term survivorship. Two studies reported that even though HDL-C levels tend to return to normal after completion of treatment for childhood ALL, the lipid profile remained abnormal for some patients [58, 61]. The three studies that have assessed blood lipids in the short term after treatment of various pediatric cancers (< 5 years on average) reported a prevalence of low HDL-C ranging from 13.1% to 42.3% which is consistent with the 16% overall prevalence found in our study [62–64]. More recently, a study including 27 pediatric patients undergoing hematopoietic stem cell transplantation, of which 17 had a neoplastic disease, also found a decrease in the prevalence of dyslipidemia 6 months after engraftment [65]. Of note, the prevalence of dyslipidemia was considerably elevated in this cohort, as 68% of children (*n* = 19/27) had dyslipidemia 6 months post-HSCT compared with 86% (*n* = 24/27) before transplantation [65].

We found that almost half of the adolescent group had low HDL-C, whereas no children were affected. In our cohort, the proportion of adolescents with low HDL-C (47.1%) is higher than what has been reported in the Canadian adolescent population (19%) [66] based on national data survey, but is comparable to male adolescent long-term (> 5 years) survivors of childhood ALL (40%) [24]. Besides, we observed lower mean plasma concentration of HDL-C and Apo A-I in adolescents compared to children. Accordingly, it has been demonstrated that lipid and lipoprotein metabolism varies with age and puberty [67, 68]. In national studies, adolescence and advanced pubertal stage were independently related to lower HDL-C concentrations compared to prepubescent children [51, 67, 69, 70]. This phenomenon has been explained by the physiological insulin resistance that occurs during puberty [67]. Being overweight or obese is another factor associated with lower HDL-C levels and lipid disorders in the general population [71, 72]. Although the difference was not statistically significant in our study, a greater proportion of adolescents than children were overweight or obese. Given the changes in body composition and hormones associated with puberty, it is possible that adolescents have a greater susceptibility than children to develop lipid anomalies early after pediatric cancer treatment.

Of the three therapeutic agents (doxorubicin, corticosteroids, methotrexate) analyzed, only doses doxorubicin influenced HDL-C and Apo A-I concentrations and the risk of having low HDL-C. Even if lipid disorders have been reported in association with doxorubicin administration [42, 43], the mechanistic cause of these disturbances remains unexplained. In vitro, in liver cells (HepG2), exposure to doxorubicin reduced ATP-binding cassette protein A1 (ABCA1) mRNA transcript levels and protein concentrations [43]. ABCA1 is a key protein in cholesterol efflux and reverse cholesterol transport. By binding to Apo A-I, it allows the transfer of CL and PL from peripheral cells to nascent lipid-poor HDL (pre-β-HDL) [73]. Furthermore, compared with untreated cells, HepG2 cells exposed to doxorubicin had lower cellular Apo A-I content, resulting into a 20% decrease in Apo A-I-mediated hepatic cholesterol efflux capacity [43]. Further studies are needed to understand the mechanism underlying the impact of doxorubicin on HDL-C and Apo A-I levels. Of note, in our cohort, while more children than adolescents had received higher doses of methotrexate and corticosteroids, none had low HDL. Therefore, analyses for these molecules were only performed in the adolescent sub-group in which we found no association between low HDL-C and higher doses. The small sample size of the adolescent sub-group may have precluded from observing associations.

In our study, evaluating total lipid and protein content of HDL3 and HDL2 revealed mainly that both HDL fractions of hypertriglyceridemic patients were enriched in TG. This was also observed in HDL3 fractions of adolescents and of patients who had received doses of doxorubicin ≥ 90 mg/m^2^. The high TG content in HDL particles and low plasma HDL-C may be explained by the TG exchange process between TG-rich lipoproteins and HDL fractions. TG-enriched HDL particles are more prone to participate in lipid exchanges in circulation with Apo-B-containing lipoproteins through the action of CETP (cholesterol ester transfer protein) and phospholipid transfer protein [74]. TG-enriched HDL depleted of EC through the action of CETP are more rapidly removed from the circulation. Thus, TGs in HDL are catabolized by hepatic lipase resulting in the formation of small HDL particles, the release of Apo A-I and higher Apo A-I degradation [75, 76]. This leads to a decrease in the number of HDL particles in circulation, which disrupts reverse cholesterol transport [77, 78] and the antioxidant and anti-inflammatory properties of HDL [79].

Comparing the 15 patients and 15 healthy controls matched for age, weight status and lipid profile revealed no difference in plasma HDL-C levels. Nonetheless, there were several disparities in lipid content of HDL2 and HDL3. Previously, our group reported similar results in a cohort of long-term childhood ALL survivors (mean age 21.1 years, survival time 12.4 years) [24]. In both cohorts, HDL2 of cancer patients were enriched in TG and FC, suggesting that these perturbations in HDL composition are maintained over time. Of note, TG enrichment of HDL fractions was also observed in other adult populations including patients with familial hypercholesterolemia [80], heavy alcohol consumers [81], and adults with type 2 diabetes and metabolic syndrome [82]. In these populations, HDL TG content was associated with the circulating levels of TG [80–82]. Accordingly, in our cohort, participants with hypertriglyceridemia had the highest mean TG content in HDL2 and HDL3. We also found that compared with healthy controls, the percentage of Apo A-I content in HDL fractions was higher whereas Apo A-II was lower in patients. This is different than what we have reported in long-term survivors, where HDL2 contained lower Apo A-I and higher Apo A-II (leading to a depressed Apo A-I/A-II ratio) compared to healthy controls [24]. It is known that the distribution of Apos in HDL varies, as some particles contain only Apo A-I, while most of the HDL particles contain both Apo A-I and Apo A-II [83]. Further studies need to confirm these findings and to assess the longitudinal evolution of HDL composition in CCS.

### Strengths and limitations

Strengths of this study include the novelty of describing HDL profile and composition early after the end of pediatric cancer treatment in relation to patients’ characteristics. In comparison to only measuring HDL-C levels, analyzing HDL content provides better insight into lipoprotein metabolism disturbances. Also, the presence of a control group allows a better appreciation of the magnitude of the changes observed in HDL particles. Our study has some limitations. Major limitations include the small sample size, the heterogeneity of the cohort and the numerous variables assessed, which reduced the likelihood of reaching the statistical significance threshold for certain analyses. Also, only three therapeutic agents were analyzed individually, whereas treatments for pediatric cancers include several other molecules in combination. Therefore, the observed derangements in lipid metabolism could have been influenced by other factors such as the type of cancer, other therapeutic agents, doses of treatment and diet. Assessing the impact of these confounding variables on the outcomes was not feasible in our study, a limitation that needs to be considered when interpreting the results. Finally, the cross-sectional design of our study does not allow for monitoring changes in HDL profile and composition parameters longitudinally after treatments.

## Conclusions

This study highlights the presence of abnormalities in plasma HDL-C and in HDL2 and HDL3 particles lipid and Apo content shortly after the end of cancer treatment. Age, overweight or obesity and doses of doxorubicin were identified as factors influencing HDL-C metabolism in this population. Given the increased risk of cardiometabolic complications and the important role of HDL in cardiovascular health, HDL characterization could allow for early identification of patients at risk once cancer treatments are terminated.

## Supplementary Information

**Additional file 1: Supplementary Table 1. **Clinical characteristics of post-treatment pediatric cancer patients: comparison based on age group at diagnosis.

**Additional file 2: Supplementary Table 2. **Plasma lipid profile of post-treatment pediatric cancer patients: comparison based on age group at diagnosis.

**Additional file 3: Supplementary Table 3. **Composition of HDL2 and HDL3 of post-treatment pediatric cancer patients: comparison based on age group at diagnosis.

## Data Availability

The datasets that support the findings of this study are available from the corresponding author upon reasonable request.

## Abbreviations

ABCA1: ATP-binding cassette protein A1
ALL: Acute lymphoblastic leukemia
Apo: Apolipoprotein
BMI: Body mass index
CCS: Long-term childhood cancer survivors
CETP: Cholesterol ester transfer protein
EC: Ester cholesterol
FC: Free cholesterol
HDL: High density lipoprotein
HDL-C: High density lipoprotein cholesterol
LCAT: Lecithin-cholestérol acyltransferase
LDL: Low density lipoprotein
LDL-C: Low density lipoprotein cholesterol
PL: Phospholipid
PR: Protein
SEM: Standard error of mean
TG: Triglycerides
