# Supplementary material for: Abnormal HDL lipid and protein composition following pediatric cancer treatment: an associative study
Source: Lipids Health Dis. 2023 Jun 10;22:72. doi: 10.1186/s12944-023-01822-2 (PMC10257312; doi:10.1186/s12944-023-01822-2)
Supplement: Supplementary file 1 — Additional file 1: Supplementary Table 1. Clinical characteristics of post-treatment pediatric cancer patients: comparison based on age group at diagnosis. [file 12944_2023_1822_MOESM1_ESM.docx]

|  | **Age group at diagnostic** | |  |  |
| --- | --- | --- | --- | --- |
| **Characteristics** | **Children (< 10 y)** | **Adolescents (≥ 10 y)** | ***p*-value** | |
|  | n (%) = 33 (66) | n (%) = 17 (34) |  | |
| **Sex, n (%)** |  |  |  | |
| Male | 12 (36.4) | 7 (41.2) | 0.767 | |
| Female | 21 (63.6) | 10 (58.8) |  | |
| **Age at evaluation, y** |  |  |  | |
| Mean ± SEM | 7.97 ± 0.37 | 17.65 ± 0.48 | **< 0.001** | |
| Median (Min-Max) | 7.75 (4.73 – 13.92) | 17.54 (13.29 – 20.92) |  | |
| **Time since diagnosis, y** |  |  |  | |
| Mean ± SEM | 3.18 ± 0.11 | 2.85 ± 0.15 | 0.101 | |
| Median (Min-Max) | 3.15 (2.15 – 4.29) | 2.75 (2.08 – 3.92) |  | |
| **Time since end of treatment, y** |  |  |  | |
| Mean ± SEM | 1.38 ± 0.14 | 1.66 ± 0.22 | 0.256 | |
| Median (Min-Max) | 1.35 (0.18 – 3.05) | 1.74 (0.23 – 3.53) |  | |
| **Treatment duration, y** |  |  |  | |
| Mean ± SEM | 1.80 ± 0.12 | 1.18 ± 0.20 | **0.031** | |
| Median (Min-Max) | 2.09 (0.20 – 3.18) | 0.99 (0.20 – 2.17) |  | |
| **Diagnosis, n (%)** |  |  |  | |
| ALL^a^ | 23 (69.7) | 4 (23.5) | **< 0.001** | |
| Lymphoma^b^ | 2 (6.1) | 9 (52.9) |  | |
| Other^c^ | 8 (24.2) | 4 (23.5) |  | |
| **Doxorubicin dose, mg/m^2^** |  |  |  | |
| Mean ± SEM | 93.7 ± 14.3 | 252.1 ± 23.9 | **< 0.001** | |
| Median (Min-Max) | 60.0 (0 – 302.0) | 255.0 (0 – 450.0) |  | |
| Doxorubicin < 90 mg/m^2^, n (%) | 24 (72.7) | 1 (5.9) | **< 0.001** | |
| Doxorubicin ≥ 90 mg/m^2^, n (%) | 9 (27.3) | 16 (94.1) |  | |
| **Corticosteroid dose, mg/m^2^** |  |  |  | |
| Mean ± SEM | 6,589 ± 708 | 4,676 ± 1,274 | 0.600 | |
| Median (Min-Max) | 8,080 (0 – 12,680) | 1,400 (0 – 12,080) |  | |
| Corticosteroids < 8,080 mg/m^2^, n (%) | 9 (27.3) | 11 (64.7) | **0.015** | |
| Corticosteroids ≥ 8,080 mg/m^2^, n (%) | 24 (72.7) | 6 (35.3) |  | |
| **Methotrexate dose, mg/m^2^** |  |  |  | |
| Mean ± SEM | 5,616 ± 597 | 2,543 ± 951 | **0.001** | |
| Median (Min-Max) | 7,710 (0 – 8,160) | 0 (0 – 12,000) |  | |
| Methotrexate < 7,230 mg/m2, n (%) | 9 (27.3) | 12 (70.6) | **0.006** | |
| Methotrexate ≥ 7,230 mg/m2, n (%) | 24 (72.7) | 5 (29.4) |  | |
| **Radiotherapy, n (%)** |  |  |  | |
| Yes | 7 (21.2) | 5 (29.4) | 0.728 | |
| No | 26 (78.8) | 12 (70.6) |  | |
| **BMI-for-age, z-score** |  |  |  | |
| Mean ± SEM | 0.30 ± 0.17 | 0.83 ± 0.36 | 0.279 | |
| Median (min-max) | 0.21 (-1.37 – 2.41) | 0.62 (-1.33 – 3.49) |  | |
| **Overweight or obese, n (%)** |  |  |  | |
| Yes | 8 (24.2) | 7 (41.2) | 0.329 | |
| No | 25 (75.8) | 10 (58.8) |  | |

**Supplementary Table 1. Clinical characteristics of post-treatment pediatric cancer patients: comparison based on age group at diagnosis**

Participants were stratified according to age at diagnosis (children: < 10 years old and adolescents: ≥ 10 years old). Pearson Chi-square (sex, doxorubicin ≥ 90 mg/m^2^, corticosteroids ≥ 8,080 mg/m^2^, methotrexate ≥ ,230 mg/m^2^, overweight or obese), Fisher exact (diagnosis, radiotherapy) and Mann-Whitney tests (age, time since diagnosis, time since end of treatment, treatment duration, doxorubicin dose mg/m^2^, corticosteroid dose mg/m^2^, methotrexate dose mg/m^2^, BMI-for-age) were performed to compare children and adolescents.

^a^Includes acute lymphoblastic leukemia (n = 22 in children and n = 4 in adolescents) and B mature leukemia with mixed-lineage leukemia (n = 1 in children).

^b^Includes non-Hodgkin lymphoma (n = 1 in children and n = 4 in adolescents) and Hodgkin’s lymphoma (n = 1 in children and n = 5 in adolescents).

^c^Other diagnoses in children include: rhabdomyosarcoma (n = 1), Wilm’s tumor (n = 2), neuroblastoma (n = 2), synovial sarcoma (n = 1), medulloblastoma (n = 1) and histiocytosis (n = 1); and in adolescents include: Ewing’s sarcoma (n = 1), rhabdomyosarcoma (n = 1), oligodendroglioma (n = 1), osteosarcoma (n = 1).

ALL: acute lymphoblastic leukemia; SEM: standard error of the mean; y: years
