# Supplementary material for: Abnormal HDL lipid and protein composition following pediatric cancer treatment: an associative study
Source: Lipids Health Dis. 2023 Jun 10;22:72. doi: 10.1186/s12944-023-01822-2 (PMC10257312; doi:10.1186/s12944-023-01822-2)
Supplement: Supplementary file 2 — Additional file 2: Supplementary Table 2. Plasma lipid profile of post-treatment pediatric cancer patients: comparison based on age group at diagnosis. [file 12944_2023_1822_MOESM2_ESM.docx]

|  | **Age group at diagnosis** | |  |
| --- | --- | --- | --- |
| **Parameters** | **Children (< 10 y)** | **Adolescents (≥ 10 y)** | ***P*-value** |
| **Dyslipidemia, n (%)** |  |  | **< 0.001** |
| Yes | 4 (12.1) | 11 (64.7) |  |
| No | 29 (87.9) | 6 (35.3) |  |
| **Cholesterol total** |  |  |  |
| **mmol/L** | n = 33 | n = 17 | 0.773 |
| Mean ± SEM | 4.09 ± 0.11 | 4.13 ± 0.24 |  |
| Median (Min-Max) | 4.00 (2.94 – 5.58) | 4.14 (2.27 – 6.09) |  |
| **z-score** | n = 28 | n = 17 | 0.454 |
| Mean ± SEM | -0.13 ± 0.18 | 0.02 ± 0.31 |  |
| Median (Min-Max) | -0.31 (-1.58 – 1.96) | 0.16 (-2.62 – 2.24) |  |
| **Hypercholesterolemia, n (%)** |  |  | 1.000 |
| Yes | 3 (9.1) | 2 (11.8) |  |
| No | 30 (90.9) | 15 (88.2) |  |
| **TG** |  |  |  |
| **mmol/L** | n = 33 | n = 17 | **< 0.001** |
| Mean ± SEM | 0.65 ± 0.04 | 1.15 ± 0.13 |  |
| Median (Min-Max) | 0.65 (0.25 – 1.15) | 0.88 (0.50 – 2.34) |  |
| **z-score** | n = 28 | n = 17 | **0.018** |
| Mean ± SEM | -0.47 ± 0.18 | 0.38 ± 0.28 |  |
| Median (Min-Max) | -0.38 (-2.71 – 1.06) | -0.01 (-1.50 – 2.42) |  |
| **Hypertriglyceridemia, n (%)** |  |  | **0.014** |
| Yes | 1(3.0) | 5 (29.4) |  |
| No | 31 (97.0) | 12 (70.6) |  |
| **LDL-C, mmol/L** | n = 33 | n = 17 | 0.487 |
| Mean ± SEM | 2.34 ± 0.11 | 2.46 ± 0.21 |  |
| Median (Min-Max) | 2.35 (1.26 – 3.74) | 2.41 (0.60 – 4.23) |  |
| **High LDL-C, n (%)** |  |  | 1.000 |
| Yes | 3 (9.1) | 2 (11.8) |  |
| No | 30 (90.9) | 15 (88.2) |  |
| **HDL-C** |  |  |  |
| **mmol/L** | n = 33 | n = 17 | **< 0.001** |
| Mean ± SEM | 1.45 ± 0.04 | 1.15 ± 0.06 |  |
| Median (Min-Max) | 1.43 (1.03 – 2.01) | 1.05 (0.85 – 1.69) |  |
| **z-score** | n = 28 | n = 17 | **0.002** |
| Mean ± SEM | 0.16 ± 0.15 | -0.64 ± 0.20 |  |
| Median (Min-Max) | -0.07 (-1.43 – 1.88) | -0.73 (-2.13 – 1.01) |  |
| **Low HDL-C, n (%)** |  |  | **< 0.001** |
| Yes | 0 (0.0) | 8 (47.1) |  |
| No | 33 (100) | 9 (52.9) |  |
| **Apo A-I, mmol/L** | n = 33 | n = 17 | **0.002** |
| Mean ± SEM | 1.44 ± 0.03 | 1.28 ± 0.06 |  |
| Median (Min-Max) | 1.42 (1.20 – 1.91) | 1.20 (0.98 – 1.75) |  |

**Supplementary Table 2. Plasma lipid profile of post-treatment pediatric cancer patients: comparison based on age group at diagnosis**

Participants were stratified according to age at diagnosis (children: < 10 years old and adolescents: ≥ 10 years old). Dyslipidemia was defined when participants presented at least one of three factors: high LDL-C, high TG, and/or low HDL-C. In children 0 to 9 years old, TG > 1.12 mmol/L, in adolescents 10 to 19 years old, TG > 1.46 mmol/L and in participants 20 – 24 years old, TG > 1.69 mmol/L were considered high. For LDL-C, values of ≥ 3.36 mmol/L in participants (0 – 19 years old) and values ≥ 4.4 mmol/L in > 19 years old were considered high. Levels of HDL-C < 1.03 mmol/L in all participants were considered low. Pearson Chi-square (dyslipidemia), Fisher exact (hypercholesterolemia, hypertriglyceridemia, high LDL-C and low HDL-C) and Mann-Whitney tests (cholesterol total, TG, LDL-C, HDL-C) were performed to compare children and adolescents. SEM: standard error of the mean; y: year.
