# Supplementary material for: Abnormal HDL lipid and protein composition following pediatric cancer treatment: an associative study
Source: Lipids Health Dis. 2023 Jun 10;22:72. doi: 10.1186/s12944-023-01822-2 (PMC10257312; doi:10.1186/s12944-023-01822-2)
Supplement: Supplementary file 3 — Additional file 3: Supplementary Table 3. Composition of HDL2 and HDL3 of post-treatment pediatric cancer patients: comparison based on age group at diagnosis. [file 12944_2023_1822_MOESM3_ESM.docx]

**Supplementary Table 3. Composition of HDL2 and HDL3 of post-treatment pediatric cancer patients: comparison based on age group at diagnosis**

|  | **TG** | **FC** | **EC** | **PL** | **PR** | **Weight ratio** [(TG+EC):(FC+PL+PR)] | |
| --- | --- | --- | --- | --- | --- | --- | --- |
| **Children (< 10 y)** | | | | | | |  |
| **HDL2** (1.125 g/mL) |  |  |  |  |  |  | |
| Total, n = 33 | 2.33 ± 0.25 | 4.87 ± 0.21 | 13.76 ± 0.61 | 29.29 ± 0.49 | 49.75 ± 0.65 | 0.19 ± 0.01 | |
| No dyslipidemia, n = 29 | 2.40 ± 0.28 | 4.92 ± 0.24 | 13.58± 0.69 | 29.10 ± 0.54 | 50.00 ± 0.72 | 0.19± 0.01 | |
| Dyslipidemia, n = 4 | 1.83 ± 0.18 | 4.54 ± 0.10 | 15.02 ± 0.41 | 30.67 ± 1.00 | 47.94 ± 0.79 | 0.20 ± 0.01 | |
| Low HDL-C, n = 0 | - | - | - | - | - | - | |
| Hypertriglyceridemia, n = 1 | 2.16 ± 0.00 | 4.35 ± 0.00 | 15.39 ± 0.00 | 30.99 ± 0.00 | 47.12 ± 0.00 | 0.21 ± 0.00 | |
| **HDL3** (1.21 g/mL) |  |  |  |  |  |  | |
| Total, n = 33 | 1.42 ± 0.12 | 1.83 ± 0.07 | 10.65 ± 0.35 | 23.87± 0.74 | 62.23 ± 0.98 | 0.14 ± 0.00 | |
| No dyslipidemia, n = 29 | 1.43 ± 0.13 | 1.85 ± 0.08 | 10.48 ± 0.38 | 23.67± 0.83 | 62.56 ± 1.09 | 0.14 ± 0.01 | |
| Dyslipidemia, n = 4 | 1.33± 0.16 | 1.69 ± 0.24 | 11.90 ± 0.65 | 25.25 ± 1.07 | 59.83 ± 1.34 | 0.15 ± 0.01 | |
| Low HDL-C, n = 0 | - | - | - | - | - | - | |
| Hypertriglyceridemia, n = 1 | 1.73 ± 0.00 | 1.17 ± 0.00 | 11.02 ± 0.00 | 22.96 ± 0.00 | 63.12 ± 0.00 | 0.15 ± 0.00 | |
| **Adolescents (≥ 10 y)** | | | | | | |  |
| **HDL2** (1.125 g/mL) |  |  |  |  |  |  | |
| Total, n = 17 | 3.33 ± 0.51 | 5.30 ± 0.35 | 11.41 ± 0.99 | 32.14 ± 2.19 | 47.82 ± 1.61 | 0.18 ± 0.02 | |
| No dyslipidemia, n = 6 | 2.60 ± 0.36 | 4.71 ± 0.46 | 12.77 ± 1.74 | 29.80 ± 2.50 | 50.13 ± 1.34 | 0.18 ± 0.03 | |
| Dyslipidemia, n = 11 | 3.72 ± 0.75 | 5.62 ± 0.46 | **10.68 ± 1.21^*^** | 33.43 ± 3.11 | 46.56 ± 2.34 | 0.17 ± 0.02 | |
| Low HDL-C, n = 8 | 2.92 ± 0.83 | 5.53 ± 0.48 | 11.55 ± 1.37 | 34.60 ± 4.26 | 45.41 ± 3.11 | 0.17 ± 0.02 | |
| Hypertriglyceridemia, n = 5 | 5.28 ± 1.09 | 5.44 ± 0.78 | 8.62 ± 1.40 | 32.89 ± 3.56 | 47.77 ± 2.37 | 0.16 ± 0.03 | |
| **HDL3** (1.21 g/mL) |  |  |  |  |  |  | |
| Total, n = 17 | **2.04 ± 0.20^**^** | 1.73 ± 0.21 | 11.57 ± 0.62 | 24.47 ± 0.76 | 60.19 ± 1.04 | **0.16 ± 0.01^*^** | |
| No dyslipidemia, n = 6 | 1.63 ± 0.19 | 1.28 ± 0.25 | **12.82 ± 1.06**^*^ | 24.48 ± 1.55 | 59.79 ± 2.34 | **0.17 ± 0.01^*^** | |
| Dyslipidemia, n = 11 | 2.26 ± 0.27 | 1.98 ± 0.28 | 10.88 ± 0.71 | 24.47 ± 0.87 | 60.41 ± 1.08 | 0.15 ± 0.01 | |
| Low HDL-C, n = 8 | 1.85 ± 0.21 | 1.87 ± 0.17 | 11.84 ± 0.39 | 24.43 ± 1.17 | 60.01 ± 1.38 | 0.16 ± 0.01 | |
| Hypertriglyceridemia, n = 5 | 3.02 ± 0.25 | 2.01 ± 0.61 | 9.54 ± 1.29 | 24.88 ± 0.61 | 60.55 ± 1.11 | 0.14 ± 0.02 | |

Data (mean ± SEM) are expressed as percentage of total HDL2 and HDL3 content of (n = 33) children and (n = 17) adolescents post-treatment pediatric cancer patients. Participants were stratified according to age at diagnosis (children: < 10 years old and adolescents: ≥ 10 years old). Children and adolescents were stratified in two groups according to their dyslipidemia status as described in Materials and Methods. Two additional subgroups were stratified among dyslipidemic children and adolescents: individuals with low HDL (children, n = 0; adolescents, n = 8) and hypertriglyceridemic individuals (children, n = 1; adolescents, n = 5). Mann-Whitney tests were performed to compare children and adolescents .**p* < 0.05, ***p* < 0.01, ****p* < 0.001 vs. children. FC: free cholesterol; EC: esterified cholesterol; PL: phospholipids; PR, protein; SEM: standard error of the mean.
